# Effectiveness and Cardiac Safety of Bedaquiline-Based Therapy for Drug-Resistant Tuberculosis: A Prospective Cohort Study

**DOI:** 10.1093/cid/ciab335

**Published:** 2021-04-21

**Authors:** James C M Brust, Neel R Gandhi, Sean Wasserman, Gary Maartens, Shaheed V Omar, Nazir A Ismail, Angela Campbell, Lindsay Joseph, Alexandria Hahn, Salim Allana, Alfonso C Hernandez-Romieu, Chenshu Zhang, Koleka Mlisana, Charle A Viljoen, Benjamin Zalta, Ismaeel Ebrahim, Meghan Franczek, Iqbal Master, Limpho Ramangoaela, Julian te Riele, Graeme Meintjes

**Affiliations:** 1 Division of General Internal Medicine, Department of Medicine, Albert Einstein College of Medicine & Montefiore Medical Center, Bronx, New York, USA; 2 Departments of Epidemiology & Global Health, Rollins School of Public Health, Emory University, Atlanta, Georgia, USA; 3 Division of Infectious Diseases, Department of Medicine, Emory School of Medicine, Emory University, Atlanta, Georgia, USA; 4 Wellcome Centre for Infectious Diseases Research in Africa, Institute of Infectious Disease and Molecular Medicine, and Department of Medicine, University of Cape Town, Cape Town, South Africa; 5 Division of Clinical Pharmacology, Department of Medicine, University of Cape Town, Cape Town, South Africa; 6 Centre for Tuberculosis, National Institute for Communicable Diseases, Johannesburg, South Africa; 7 Department of Molecular Medicine & Haematology, School of Pathology, Faculty of Health Sciences, University of the Witwatersrand, Johannesburg, South Africa; 8 National Health Laboratory Services, Johannesburg, South Africa; 9 Division of Cardiology, Department of Medicine, University of Cape Town, Cape Town, South Africa; 10 Department of Radiology, Albert Einstein College of Medicine & Montefiore Medical Center, Bronx, New York, USA; 11 King Dinuzulu Hospital Complex, Durban, South Africa; 12 Jose Pearson Hospital, Port Elizabeth, South Africa; 13 Brooklyn Chest Hospital, Cape Town, South Africa

**Keywords:** bedaquiline, multidrug-resistant tuberculosis, extensively drug-resistant tuberculosis, QT interval, HIV, clofazimine, antiretroviral therapy

## Abstract

**Background:**

Bedaquiline improves treatment outcomes in patients with rifampin-resistant (RR) tuberculosis but prolongs the QT interval and carries a black-box warning from the US Food and Drug Administration. The World Health Organization recommends that all patients with RR tuberculosis receive a regimen containing bedaquiline, yet a phase 3 clinical trial demonstrating its cardiac safety has not been published.

**Methods:**

We conducted an observational cohort study of patients with RR tuberculosis from 3 provinces in South Africa who received regimens containing bedaquiline. We performed rigorous cardiac monitoring, which included obtaining electrocardiograms in triplicate at 4 time points during bedaquiline therapy. Participants were followed up until the end of therapy or 24 months. Outcomes included final tuberculosis treatment outcome and QT interval prolongation (QT prolongation), defined as any QT interval corrected by the Fridericia method (QTcF) >500 ms or an absolute change from baseline (ΔQTcF) >60 ms.

**Results:**

We enrolled 195 eligible participants, of whom 40% had extensively drug-resistant tuberculosis. Most participants (97%) received concurrent clofazimine. Of the participants, 74% were cured or successfully completed treatment, and outcomes did not differ by human immunodeficiency virus status. QTcF continued to increase throughout bedaquiline therapy, with a mean increase (standard deviation) of 23.7 (22.7) ms from baseline to month 6. Four participants experienced a QTcF >500 ms and 19 experienced a ΔQTcF >60 ms. Older age was independently associated with QT prolongation. QT prolongation was neither more common nor more severe in participants receiving concurrent lopinavir-ritonavir.

**Conclusions:**

Severe QT prolongation was uncommon and did not require permanent discontinuation of either bedaquiline or clofazimine. Close monitoring of the QT interval may be advisable in older patients.

## Teaser

Severe prolongation of the QT interval corrected by the Fridericia method (QTcF) was uncommon among participants treated for multidrug-resistant or extensively drug-resistant tuberculosis with a regimen containing both bedaquiline and clofazimine. Outcomes were favorable in this setting with high human immunodeficiency virus prevalence. Participants receiving concurrent lopinavir-ritonavir did not experience further QTcF prolongation.

Drug-resistant tuberculosis remains a major public health threat and undermines control of tuberculosis worldwide. Bedaquiline was the first antituberculosis drug from a novel class to be approved in more than 40 years [1]; its approval by the Food and Drug Administration (FDA) and the European Medicines Agency was based on results of 3 small phase 2 trials [2–4]. Observational data have shown improved treatment outcomes in patients with multidrug-resistant (MDR) or extensively drug-resistant (XDR) tuberculosis who received regimens containing bedaquiline [5, 6]. Based on these data, the World Health Organization (WHO) recommends that all patients with rifampin-resistant (RR) tuberculosis be treated with a regimen containing bedaquiline [7].

Bedaquiline prolongs the QT interval, resulting in concerns about its cardiac safety [8]. In the pivotal phase 2 trial, there were more deaths in the bedaquiline arm compared with placebo [3, 9]. Although the deaths were not directly attributed to bedaquiline [10], the FDA created a “black box” warning about excess mortality and QT interval prolongation (QT prolongation) [11], and guidelines advise electrocardiographic monitoring in patients receiving bedaquiline [7]. A systematic review of the cardiac safety of bedaquiline reported wide variability in QT prolongation observed, but most included studies were retrospective, based on electrocardiograms (ECGs) obtained in routine clinical practice, and patients were receiving other QT-prolonging drugs [12]. To date, there are still no phase 3 trial data assessing bedaquiline’s cardiac safety. WHO recommends that bedaquiline be given with clofazimine, based on improved outcomes in observational studies [7], but clofazimine also prolongs the QT interval [13], resulting in additive QT prolongation when combined with bedaquiline [4].

A meta-analysis of MDR tuberculosis cohort studies found that human immunodeficiency virus (HIV) coinfection more than doubled the adjusted odds of death [14]. Treatment with concurrent antiretroviral therapy (ART) improves these outcomes considerably [15] and is recommended in all patients with drug-resistant tuberculosis and HIV coinfection [7]. However, there are important drug-drug interactions between bedaquiline and some antiretrovirals: efavirenz is contraindicated as it induces bedaquiline metabolism, decreasing bedaquiline concentrations, and [16] lopinavir-ritonavir inhibits bedaquiline metabolism, resulting in a nearly 2-fold increase in bedaquiline exposure [17]; the clinical importance of this interaction is not known. We determined the effectiveness and cardiac safety (by rigorously assessing the QT interval) in patients with drug-resistant tuberculosis treated with bedaquiline in a setting with high HIV prevalence.

## METHODS

### Setting

The Pharmacokinetics, Resistance, and Outcomes of Bedaquiline in MDR and XDR-TB (PROBeX) study was a prospective observational cohort study conducted between 2016 and 2020 at 3 drug-resistant tuberculosis referral hospitals in South Africa. During the study period, all patients with pre-XDR and XDR tuberculosis, as well as patients with RR tuberculosis for whom an injectable agent was contraindicated or poorly tolerated, were treated with a modified standardized regimen, which typically included bedaquiline (400 mg/d for 2 weeks, followed by 200 mg 3 times weekly), linezolid (600 mg/d), clofazimine (100 mg/d), levofloxacin (750–1000 mg/d), ethionamide (15–20 mg/kg; maximum, 750 mg/d), terizidone (15–20 mg/kg; maximum, 750 mg/d), and pyrazinamide (20–30 mg/kg; maximum, 1600 mg/d). Bedaquiline was given for 6 months, and the total tuberculosis treatment duration was 18–24 months. Para-amino salicylic acid, high-dose isoniazid, kanamycin, amikacin, ethambutol, rifabutin, and delamanid were given to some participants at the discretion of the treating provider. 

Study team members were not directly involved in individual treatment decisions. Many participants were already receiving tuberculosis therapy before bedaquiline initiation, and those receiving a regimen containing moxifloxacin before bedaquiline initiation were changed to levofloxacin per standard of care. All HIV-coinfected participants were offered ART irrespective of CD4 cell count. Because efavirenz is contraindicated with bedaquiline, all HIV-infected participants received either nevirapine- or lopinavir-ritonavir–based ART.

### Study Population and Procedures

We recruited patients ≥18 years old with culture-confirmed tuberculosis who were starting treatment with a bedaquiline-containing regimen between April 2016 and March 2018. Eligibility for bedaquiline therapy in the national tuberculosis program required a baseline QT interval corrected by the Fridericia method (QTcF) of ≤450 ms. In addition, participants were excluded from the study if they had received bedaquiline treatment, had abnormal baseline creatinine levels (>2 times the upper limit of normal), or had abnormal alanine aminotransferase levels (>5 times the upper limit of normal). Participants had to agree to HIV testing if their HIV status was unknown.

Participants were followed up biweekly for the first 3 months of therapy, monthly for months 4–6, and then at months 12, 18 and 24, or until 6 months after the completion of therapy, whichever was earliest. At each visit, participants were interviewed regarding current symptoms and adverse events (AEs). Study ECGs were obtained by trained study staff at baseline and at months 1, 2, and 6. After participants had rested in a supine position for several minutes, 3 ECGs were obtained at each time point, ≥5 minutes apart. All QT intervals were manually measured by a single cardiologist (C.V.) and corrected using Fridericia’s formula [18]. In addition, all participants had routine safety monitoring consisting of monthly (single) ECGs obtained and read by clinic providers. 

Study staff did not review or capture clinic ECGs, and decisions to stop therapy were made by clinic providers rather than the study team. Because moxifloxacin also prolongs the QT interval, we identified participants who discontinued moxifloxacin <24 hours before their baseline ECG in the analysis. Sputum samples were sent for mycobacterial culture (Mycobacterial Growth Indicator Tube [MGIT] 960 system; Bactec) biweekly for the first 3 months, and then monthly thereafter. Bedaquiline minimum inhibitory concentrations (MICs) were measured on all available isolates, using the MGIT system [19], at the Centre for Tuberculosis in the National Institute for Communicable Diseases in Johannesburg, South Africa. Drug susceptibility testing for other drugs was performed at the regional reference tuberculosis laboratories.

### Outcome Measures and Analysis

The primary effectiveness outcome of interest was cure or treatment completion according to WHO definitions [20]. The primary safety outcome was prolongation of the QTcF interval, defined as any instance of QTcF interval >500 ms or an increase in QTcF (ΔQTcF) from baseline of >60 ms. Secondary outcomes included survival, time to tuberculosis culture conversion, development of resistance to bedaquiline, serious AEs (SAEs), and any instance of QTcF >450 ms or ΔQTcF >30 ms. Bedaquiline resistance was defined as having an MIC >1 μg/mL, as measured with the MGIT system [19]. Targeted Sanger sequencing of the *Rv0678* gene was done on isolates with phenotypic resistance to bedaquiline. 

Time to culture conversion was calculated, in days, from the date of bedaquiline initiation to the first of 2 consecutive negative cultures taken ≥4 weeks apart. HIV virologic suppression was defined as a viral load <150 copies/mL (the lower limit of detection of certain assays used during the study period). SAEs were defined as clinical events that resulted in death, hospitalization, or discontinuation of therapy, or laboratory abnormalities of grade 3 or 4 according to the Division of AIDS toxicity table [21].

Participant characteristics were compared using simple frequencies, χ ^2^ tests, and Wilcoxon rank sum tests. Survival analysis was performed using Kaplan-Meier curves and log-rank tests. The mean of the 3 QTcF values at each time point was used for comparison with those at other time points, and participants were stratified by HIV status, receipt of lopinavir-ritonavir, and concurrent use of moxifloxacin. We used generalized estimating equations to analyze the change in mean QTcF over time and logistic regression to examine clinical predictors of QTcF prolongation.

### Ethical Approval

The study was approved by the institutional review boards at the University of Cape Town, Albert Einstein College of Medicine, and Emory University. All participants signed written informed consent.

## RESULTS

**Figure 1.**
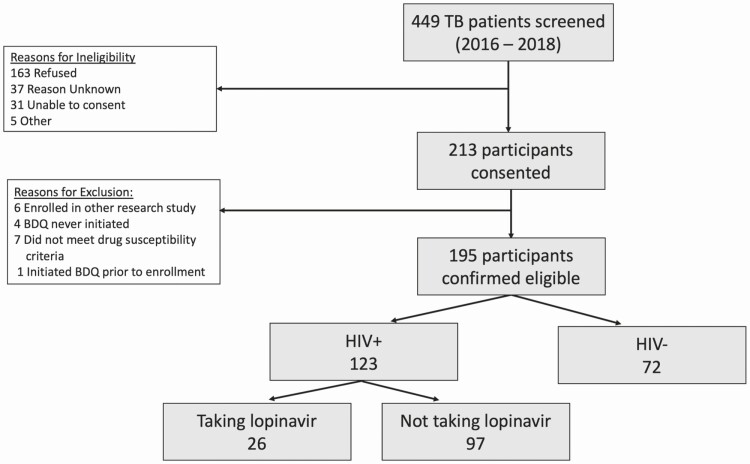
Enrollment flowchart. Abbreviations: BDQ, bedaquiline; HIV, human immunodeficiency virus.

We screened patients with presumed RR tuberculosis, of whom 195 were eligible for enrollment (Figure 1): 80 (41%) had XDR, 78 (40%) had pre-XDR, and 29 (15%) had MDR tuberculosis; 123 (63%) were HIV infected (Table 1). The median age (interquartile range [IQR]) was 33 (28–42) years, and 111 participants (57%) were female; 40% were sputum smear positive, 77% had cavitary disease, and 66% had previously had tuberculosis. Nine participants (7%) had received clofazimine before study enrollment. During the study period, 190 (97%) received concurrent clofazimine, and 179 (92%) received concurrent linezolid (Table 2). Among HIV-infected participants, the median (IQR) CD4 cell count at enrollment was 196/μL (96–427/μL). Of the HIV-infected participants, 113 (90%) were already receiving ART at the time of enrollment (median duration, 8 months). A total of 26 participants (23%) received an ART regimen containing lopinavir-ritonavir during the study; 23 started lopinavir-ritonavir before bedaquiline and 3 participants started lopinavir-ritonavir later (1–4 months after bedaquiline initiation). In only 28% of those with an available baseline viral load (28 of 100) was this undetectable.

**Table 1.** Participant Characteristics

Characteristics	Participants by Study Site, No. (%)^a^			
	All Sites (n = 195)	Durban (n = 89)	Port Elizabeth (n = 47)	Cape Town (n = 59)
Demographic				
Age, median (IQR), y	33 (28–42)	32 (27–39)	35 (30–42)	29 (26–43)
Female sex	111 (57)	55 (62)	21 (45)	35 (59)
Race				
Black	160 (82)	89 (100)	38 (81)	33 (56)
Mixed race	33 (17)	0 (0)	9 (19)	24 (41)
White	2 (1)	0 (0)	0 (0)	2 (3)
Clinical				
BMI, median (IQR)^b^	20 (18–23)	21 (18–22)	19 (17–23)	19 (18–24)
BMI group^b^				
<18	55/193 (28)	23 (26)	14 (30)	18 (32)
18–25	103/193 (53)	51 (57)	26 (55)	26 (46)
25–30	21/193 (11)	8 (9)	4 (9)	9 (16)
>30	14/193 (7)	7 (8)	3 (6)	4 (7)
HIV infected	123 (63)	66 (74)	28 (60)	29 (49)
Receiving any ART	113 (92)	66 (100)	23 (82)	24 (83)
Receiving ART regimen including lopinavir	26 (23)	11 (17)	3 (13)	12 (50)
Duration of ART at enrollment, median, mo	8	8	29.5	5
CD4 cell count at enrollment, median (IQR), cells/μL	196 (96–427)	185 (105–433)	196 (105–575)	210 (72–353)
Undetectable HIV viral load at enrollment, %	28	30	13	17
Diabetes	10 (5)	3 (3)	5 (11)	2 (3)
Current/former smoker	62 (32)	9 (10)	21 (45)	32 (54)
Alcohol use in past year	73 (37)	14 (16)	29 (62)	30 (51)
QTcF at baseline, mean (SD), ms	404.6 (22.1)	408.1 (24.0)	401.9 (20.1)	401.5 (20.2)
Tuberculosis				
Resistance category				
MDR	29 (15)	21 (24)	7 (15)	1 (2)
Pre-XDR	78 (40)	34 (38)	10 (21)	34 (58)
XDR	80 (41)	28 (31)	30 (64)	22 (37)
Other RR tuberculosis^c^	8 (4)	6 (7)	0 (0)	2 (3)
Sputum smear positive,	73/181 (40)	32/85 (38)	19/45 (42)	22 (37)
Any prior tuberculosis episode	128 (66)	57 (64)	26 (51)	45 (75)
Prior episodes, median (IQR), no.	2 (2–3)	2 (2–3)	2 (2–2)	3 (2–3)
Prior drug-susceptible tuberculosis	60^d^ (48)	18 (32)	18 (75)	23 (51)
Prior drug-resistant tuberculosis	66^d^ (52)	39 (57)	6 (25)	22 (49)
Prior treatment with CFZ	9 (7)	3 (3)	2 (8)	4 (9)
Duration of prior treatment with CFZ, median, (IQR), mo	2 (1–10.5)	3^e^	Unknown	1 (1–18)
Baseline chest radiographic findings	(n = 125)	(n = 63)	(n = 45)	(n = 17)
Cavitary lesion	96 (77)	42 (67)	40 (89)	14 (82)
Bilateral disease	71 (57)	32 (51)	28 (62)	11 (65)

Abbreviations: ART, antiretroviral therapy; BMI, body mass index; CFZ, clofazimine; HIV, human immunodeficiency virus: IQR, interquartile range; MDR, multidrug-resistant; QTcF, QT interval corrected by the Fridericia method; RR, rifampin-resistant; SD, standard deviation; XDR, extensively drug-resistant.

^a^Data represent no. (%) of participants, unless otherwise specified.

^b^BMI was calculated as weight in kilograms divided by height in meters squared.

^c^Five participants had only Xpert test results, with no additional susceptibility test results.

^d^Details on previous treatment were available for 126 participants.

^e^The duration of prior clofazimine treatment was unknown for 2 of the 3 participants.

**Table 2.** Antituberculosis Drugs Received After Enrollment

Drug	Participants Receiving Drug, No. (%)
Bedaquiline	195 (100)
Clofazimine	190 (97)
Pyrazinamide	184 (94)
Levofloxacin	183 (94)
Linezolid	179 (92)
Para-aminosalicylic acid	173 (89)
Terizidone	161 (83)
Ethambutol	93 (48)
Moxifloxacin^a^	49 (25)
High-dose isoniazid	74 (38)
Ethionamide	63 (32)
Kanamycin or amikacin	16 (8)
Delamanid	11 (6)
Rifabutin	5 (3)

^a^Of the 49 participants receiving moxifloxacin, 32 received it concurrently with bedaquiline for ≥24 hours; 40 of the 49 were changed from levofloxacin to moxifloxacin after completing the 6-month course of bedaquiline.

### Tuberculosis Treatment Outcomes

**Figure 2.**
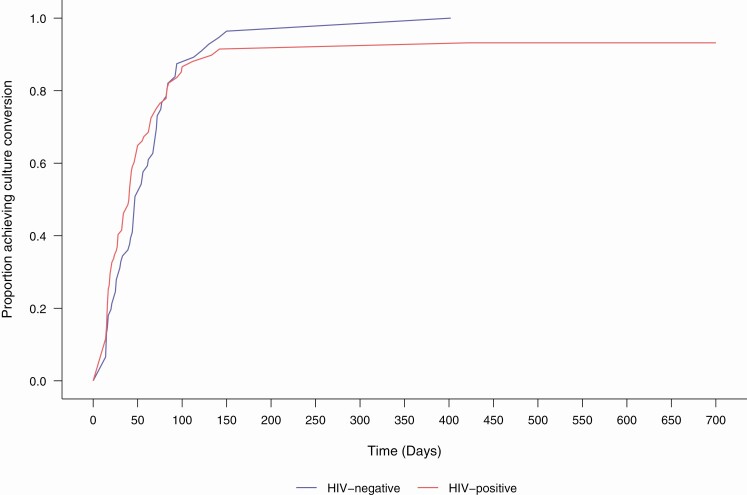
Kaplan-Meier plot of time to sputum culture conversion among participants with positive culture at time of bedaquiline initiation (n = 158), stratified by human immunodeficiency virus (HIV) status.

Participants were followed up for a median (IQR) of 22 (14–24) months after starting bedaquiline (300 person-years). Among the 195 enrolled participants, sputum culture conversion was achieved in 174 (89%), before bedaquiline initiation in 37 (19%). Among the 137 (70%) with culture conversion after bedaquiline was started, the median (IQR) time to conversion was 41 (17–67) days (Figure 2). Among all participants, 145 (74%) had a successful tuberculosis treatment outcome (cure in 129 [66%]; treatment completed in 16 [8%]). Eight participants (4%) experienced treatment failure, 18 (9%) interrupted treatment prematurely, and 25 (13%) died (Supplementary Appendix Table 1). The proportion of participants with treatment success did not significantly differ by resistance category (69% for MDR vs 77% for pre-XDR vs 74% for XDR tuberculosis; *P* = .67). Among participants who died, the median survival time (IQR) was 2.3 (1.1–6.8) months (Supplementary Figure 1). Treatment outcomes did not differ between those with and those without HIV (*P* = .61).

### QTcF Prolongation

**Figure 3.**
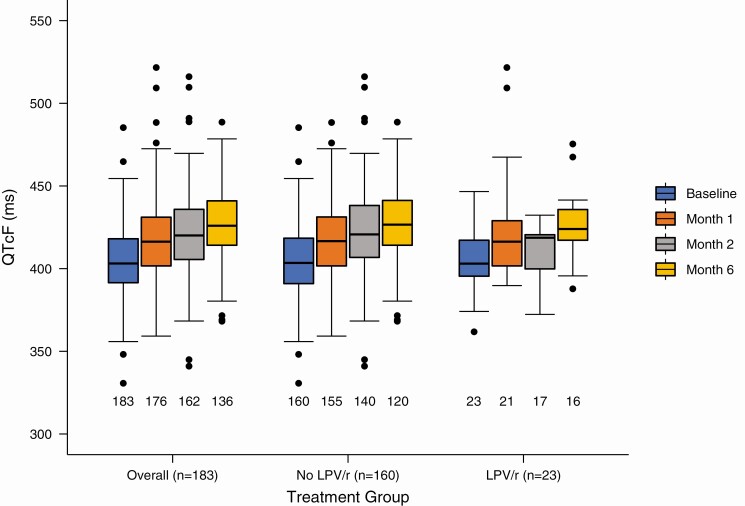
QT interval corrected by the Fridericia method (QTcF) from baseline to month 6 overall and stratified by receipt of lopinavir-ritonavir (LPV/r). Numbers below plots represent the number of available paired electrocardiograms at each visit.

One hundred eighty-three (94%) participants had ≥1 baseline (before bedaquiline) and 1 follow-up ECG and were included in the electrocardiographic analysis. The mean (SD) QTcF at baseline was 404.6 (22.1) ms (Table 3). One hundred twenty (66%) participants received moxifloxacin during the study or immediately before study enrollment. Of these, 99 (83%) stopped moxifloxacin before starting bedaquiline, ≥24 hours before starting it in 70 (58%). The median moxifloxacin washout period was 1 day (IQR, 1–2 days). The mean (SD) maximum QTcF for all participants was 434.4 (24.5) ms. Among participants receiving clofazimine (n = 179), the mean (SD) maximum QTcF was 434.8 (24.4) ms, compared with 416.7 (27.3) ms) in those not receiving clofazimine (n = 4; *P* = .15). Among participants receiving concurrent lopinavir-ritonavir, the mean (SD) maximum QTcF was 437.1 (31.0) ms, compared with 434.0 (23.5) ms in those not receiving lopinavir-ritonavir (*P* = .57). Among all participants, QTcF continued to increase while on bedaquiline, and the mean (SD) increase in QTcF from baseline to month 6 was 23.7 (22.7) ms (*P* < .001); this did not differ based on receipt of concurrent lopinavir-ritonavir (*P* = .61; Figure 3).

**Table 3.** Electrocardiographic Findings

Finding	All Patients (n = 183)	BDQ Only (n = 4)	BDQ and CFZ (n = 179)	BDQ and LPV/r (With or Without CFZ) (n = 23)	BDQ Without LPV/r (n = 160)
QTcF, mean (SD), ms					
Baseline	404.6 (22.2)	398.8 (21.1)	404.7 (22.2)	405.1 (20.3)	404.5 (22.5)
mo 1	418.7 (24.3)	403.5 (20.2)	419.1 (24.4)	425.5 (35)	417.8 (22.5)
mo 2	421.2 (25.4)	429.2 (13.6)	421.0 (25.6)	411.9 (16.8)	422.3 (26.1)
mo 6	427.6 (22.1)	…	427.6 (22.1)	427.5 (22.3)	427.6 (22.2)
Maximum QTcF, mean (SD) (all participants)	434.4 (24.5)	416.7 (27.3)	434.8 (24.4)	437.1 (31.0)	434.0 (23.5)
Participants receiving MFX before BDQ initiation, no.	117	2	115	13	104
Participants who stopped MFX before starting BDQ, no. (%)	96 (82)	2 (100)	94 (82)	11 (85)	85 (82)
Duration of MFX washout before baseline ECG, median (IQR), d	1 (0–2)	1 (1–69)	1 (0–2)	1 (0–2)	1 (0–2)
QTcF increase from baseline to mo 6, mean (SD)	23.7 (22.7)	…	23.7 (22.7)	26.4 (22.2)	23.4 (22.9)
Participants, no. (%)					
With mean QTcF increase >60 ms	8 (4.4)	0 (0)	8 (4.5)	2 (8.7)	6 (3.8)
With mean QTcF increase >30 ms	61 (33.3)	1 (25)	60 (33.5)	6 (26.1)	55 (34.4)
With QTcF >500 ms	4 (2)	0 (0)	4 (2.2)	2 (8.7)	2 (1.3)
With QTcF >450 ms	42 (23)	0 (0)	42 (23.5)	5 (21.7)	37 (23.1)

Abbreviations: BDQ, bedaquiline; CFZ, clofazimine; ECG, electrocardiogram; IQR, interquartile range; LPV/r, lopinavir-ritonavir; MFX, moxifloxacin; QTcF, QT interval corrected by the Fridericia method; SD, standard deviation.

Nineteen participants (10.4%) experienced a ΔQTcF >60 ms at any time; all 19 received concurrent clofazimine, 4 received lopinavir-ritonavir, and 2 did not have a moxifloxacin washout before bedaquiline initiation. Four participants (2.2%) experienced a QTcF >500 ms; all 4 received concurrent clofazimine, 2 received lopinavir-ritonavir, and none received moxifloxacin. After adjustment for age, race, sex, weight, receipt of lopinavir-ritonavir, and concurrent moxifloxacin, age >30 years remained significantly associated with QTcF >450 ms compared with age 21–30 years, with the greatest effect seen in those >50 years old (adjusted odds ratio, 8.3 [95% confidence interval, 2.1–32.8]; Table 4). Supplementary Table 2 shows the age strata for the participants experiencing QTcF >500 ms or ΔQTcF >60 ms.

**Table 4.** Multivariable Logistic Regression Analysis of Potential Predictors of QTcF Prolongation

Variable	aOR 95% CI	
	QTcF >450 ms	ΔQTcF >30 ms
Male sex	1.3 (.6–2.7)	1.2 (.6–2.2)
Black race	3.2 (.97–10.41)	1.5 (.7–3.2)
Age, y^a^		
21–30 (n = 45)	Reference	Reference
31–40 (n = 72)	3.4 (1.0–10.9)^b^	1.6 (.8–3.5)
41–50 (n = 43)	3.8 (1.1–13.9)^b^	1.6 (.7–3.9)
>50 (n = 23)	8.3 (2.1–32.8)^b^	1.9 (.7–5.3)
Weight (per 1-kg increase)	0.99 (.95–1.02)	0.98 (.96–1.01)
Concurrent lopinavir-ritonavir	0.82 (.3–2.6)	0.86 (.32–1.81)
Concurrent moxifloxacin^c^	1.4 (.5–3.6)	0.89 (.3–2.2)

Abbreviations: aOR, adjusted odds ratio; CI, confidence interval; QTcF, QT interval corrected by the Fridericia method.

^a^The youngest study participant was 21 years old.

^b^Significant association with age.

^c^Including participants who received moxifloxacin concurrently with bedaquiline for ≥1 day, or who discontinued moxifloxacin <24 hours before initiating bedaquiline.

### Bedaquiline Resistance

Eighty-four participants had a baseline *M. tuberculosis* isolate available for bedaquiline MIC testing. Of these, 7 (8%) had a bedaquiline MIC >1 μg/mL before initiation of therapy (range, 2–8 μg/mL; Table 5). Two additional participants had a bedaquiline MIC of 4 μg/mL at their 1-month visit, but their baseline isolates were not available for testing. Only 1 of these 9 participants had received prior clofazimine therapy.

**Table 5.** Findings in Participants Having ≥1 *Mycobacterium tuberculosis* Isolate With a Bedaquiline Minimum Inhibitory Concentration >1 μg/mL

Participant ID	Visit When Resistant Isolate Was Obtained	MIC to BDQ, μg/mL	*Rv0678*	Cavitation on Baseline Chest Radiograph	Prior CFZ	Treatment Outcome
**Resistance at baseline**						
A	Baseline	4	144insC	NA	No	Cure
B	Baseline	4	144insC	Yes	No	Interruption/LTFU
C	Baseline	4	T437C	NA	No	Cure
D	Baseline	2	WT	MA	No	Interruption/LTFU
E	Baseline	8	139_142insGATC	Yes	No	Cure
F	Baseline	4	138insG	Yes	Yes (unknown duration)	Treatment completion
G	Baseline	4	A202C	Yes	No	Death
**Emergent resistance with therapy**						
H	mo 10	8	349insC	Yes	No	Cure
I	mo 17	4	A202G	Yes	No	Death (after interruption)
J	wk 6	2	WT	Yes	Yes (unknown duration)	Interruption/LTFU
**Resistance with therapy but with no available baseline isolate**						
K	mo 6	4	141insT/139insG	NA	No	Death
L	mo 1	4	144insG	NA	No	Failure
M	mo 1	4	198insG	Yes	No	Cure

Abbreviations: BDQ, bedaquiline; CFZ, clofazimine; ID, identifier; LTFU, lost to follow-up; MIC, minimum inhibitory concentration; NA, chest radiograph not available; WT, wild type;

Four participants were found to have a bedaquiline MIC >1 μg/mL during treatment after the 1-month visit (range, 2–8 μg/mL); 3 of these participants had a baseline isolate which was susceptible, and the baseline isolate was not available for the fourth. Of the 13 participants with an elevated MIC at any time point, 6 were eventually cured, 3 interrupted therapy, 3 died, and 1 experienced treatment failure. Among these 13 participants, polymorphisms were found in *Rv0678* for 11 (85%). Only 2 of the participants had the same polymorphism, and the other 9 were unique. Among those with resistance at baseline or month 1 (n = 9), a successful outcome was achieved in 55%.

### Occurrence of SAEs

Overall, SAEs were common, with 84 participants (43%) experiencing a clinical or laboratory AE that required temporary or permanent discontinuation of ≥1 antituberculosis medications. Most discontinuations (n = 56) were due to linezolid-associated AEs, but bedaquiline was stopped in 9 participants (5%), owing to QT prolongation (n = 5), rash (n = 1), abdominal pain (n = 1), nonspecific T-wave abnormality (n = 1), and unknown cause (n = 1). Four of the 5 participants who stopped bedaquiline because of QT prolongation also stopped clofazimine, although all 4 eventually restarted both drugs, and the fifth participant restarted bedaquiline. Only 1 of these 5 participants experienced a ΔQTcF >60 ms, and none experienced a QTcF >500 ms as shown by study ECGs. The participant with rash temporarily stopped all tuberculosis medications. Four participants (2%) experienced a grade 3 or 4 elevation in ALT, which resolved spontaneously in all 4 without any discontinuation in therapy, potentially representing hepatic adaptation. Participants receiving concurrent lopinavir-ritonavir and bedaquiline were no more likely to experience clinical or laboratory SAEs than those who received other ART regimens (*P* = .61).

## DISCUSSION

In this prospective cohort study, we followed up participants with RR tuberculosis who were treated with bedaquiline to rigorously assess their treatment outcomes and cardiac safety. Treatment outcomes were generally favorable, as has been shown by others [5, 6, 22, 26]. Few participants experienced a QTcF >500 ms or an absolute increase of >60 ms from baseline, suggesting that bedaquiline, even in combination with clofazimine, may be safe. This is an important finding, given WHO’s recommendation that most patients with RR tuberculosis be treated with both bedaquiline and clofazimine. The largest increase in QTcF was at month 1, but the QTcF continued to increase for the duration of bedaquiline therapy, suggesting that it may not have plateaued when bedaquiline was stopped at 6 months. As more patients are treated with bedaquiline worldwide and, potentially with courses longer than 6 months [24, 25], QT monitoring in the later months of therapy will be important to ensure that the QTcF does not reach dangerous levels. Older age, particularly >50 years, was associated with QT prolongation and may warrant close cardiac monitoring.

Nearly all of our study participants received both bedaquiline and clofazimine. Participants treated with both bedaquiline and clofazimine had a longer QTcF at all study visits, compared with those who did not receive clofazimine, but this comparison is limited by the small number of participants who did not receive clofazimine. The QTcF among participants concurrently treated with clofazimine was also longer than the QTcF in participants in other studies who received bedaquiline without clofazimine [3, 26].

Lopinavir-ritonavir reduces bedaquiline clearance, leading to an approximately 2-fold increase in steady-state concentration [17], but until now, the clinical importance of this interaction was unknown. We found that participants who received concurrent therapy did not experience a significant prolongation in QTcF compared with those treated with bedaquiline who did not receive lopinavir-ritonavir. This is likely because lopinavir-ritonavir has a minimal effect on plasma concentrations of bedaquiline’s M2 metabolite [27], which is responsible for the QT prolongation seen with bedaquiline [28, 29].

Bedaquiline discontinuations were uncommon (5%) and frequently temporary. Although most study participants had a successful treatment outcome, 15% of the 99 participants tested for bedaquiline resistance had an elevated MIC to bedaquiline at some time point: some had resistant isolates at baseline, while in others resistance developed during therapy or after a treatment interruption. The presence of bedaquiline resistance at baseline is concerning and has been seen in other studies [30, 31]. Prior exposure to clofazimine may generate polymorphisms in *Rv0678* and cross-resistance to bedaquiline [32], but very few participants in our study had previously received clofazimine. While these variants could represent spontaneous polymorphisms, it is also possible that bedaquiline resistance is already being transmitted. We used a consensus definition of resistance based on an MIC cutoff of 1.0 μg/mL as measured with the MGIT system, but importantly, this definition was developed without clinical outcomes [19, 33]. A clinical definition of resistance is challenging in multidrug therapy, because participants may have a favorable outcome despite bedaquiline resistance if the background regimen contains a sufficient number of active drugs, as we observed in some patients in the current study.

A strength of our study is the precision with which we obtained and analyzed ECGs; ECGs were timed and obtained in triplicate, and all QTcF intervals were measured by a cardiologist. We also reported the use of moxifloxacin at baseline and the duration of the washout period, to have a more precise estimate of the incremental effect of bedaquiline (and clofazimine). 

Our study does have several limitations. First, we were reliant on self-report of AEs and clinician notes from the handwritten medical record. Some AEs may, therefore, have been incompletely captured. We restricted our analysis, however, only to SAEs—particularly those requiring a change in therapy and/or hospitalization, as these would have been unlikely to go unnoted in the medical record. Second, *Mycobacterium tuberculosis* isolates were only available from 2 of the 3 study sites. Third, participants were followed up for a maximum of 24 months, and we therefore did not capture information on relapse after treatment completion. Fourth, we used an outcome of QTcF >450 ms and ΔQTcF >30 ms from baseline in our predictors analysis owing to the small number of participants who experienced the more clinically important outcomes of QTcF >500 or ΔQTcF >60 ms. These alternate end points are approved by the FDA [34] but are not as clearly associated with sudden cardiac death. We did not test differing monitoring strategies, and we are thus unable to recommend a monitoring schedule for clinical care. Our study ECGs were obtained in triplicate and read by a cardiologist, which is important for research but is not feasible for routine monitoring.

Our findings suggest that the combination of bedaquiline and clofazimine is safe and that life-threatening QTcF prolongation is rare. Our study adds to the literature establishing the cardiac safety of bedaquiline when given with other QT-prolonging medications [35, 36], including a randomized controlled trial to evaluate the cardiac safety of concomitant bedaquiline and delamanid. An important population of HIV-infected patients worldwide will require protease inhibitor-based ART; therefore, demonstrating the safety of lopinavir-ritonavir with bedaquiline has important implications for clinical practice. In just 8 years, bedaquiline has transformed the treatment of MDR and XDR tuberculosis. Several clinical trials are currently underway to identify the optimal combination of partner medications. Defining the drug-drug interactions with bedaquiline and their clinical implications is essential in order for the tuberculosis community to optimize the use of this important drug.

## Supplementary Data

Supplementary materials are available at *Clinical Infectious Diseases* online. Consisting of data provided by the authors to benefit the reader, the posted materials are not copyedited and are the sole responsibility of the authors, so questions or comments should be addressed to the corresponding author.

ciab335_suppl_Supplementary_Materials

## Notes

***Acknowledgments.*** We are grateful to the study team at the University of Cape Town for their tireless efforts in data collection, record abstraction, participant recruitment and interviews. We thank the doctors and staff of Brooklyn Chest Hospital, Jose Pearson Hospital, and King Dinuzulu Hospital for their outstanding clinical management of the study participants and support of the study. We thank Cindy Hayes, Tania Dolby, and Keeren Lutchminarain for their assistance with obtaining laboratory specimens. We thank Pauline Harrington, Krystalyn Martin, and Zahraa Mohamed for their contributions to data cleaning and analysis, and Jonathan Smith for his assistance with the study figures. Finally, we thank the participants who consented to participate in this study, as well as their families.

***Author note.*** Coauthor Iqbal Master died on January 15, 2021.

***Financial support***. This study was funded by the National Institute of Allergy and Infectious Diseases, National Institutes of Health (grants R01AI114304 , R01AI145679, and K24AI155045 to J. C. M. B. and K24AI114444 to N. R. G.), Einstein-Rockefeller-CUNY Center for AIDS Research (grant P30AI124414), Emory Center for AIDS Research (grant P30AI050409), Emory Tuberculosis Research Unit (grant U19AI111211), Einstein/Montefiore Institute for Clinical and Translational Research (grant UL1TR001073), the Atlanta Clinical and Translational Science Institute (grant UL1TR000454), the European & Developing Countries Clinical Trials Partnership (grant CDF1018 to S. W.), Wellcome Trust (grant 203135/Z/16/Z to S. W. and grants 098316, 214321/Z/18/Z, and 203135/Z/16/Z to G. Meintjes), and the National Institutes of Health (grant K43TW011421 to S. W.), the South African Research Chairs Initiative of the Department of Science and Technology and National Research Foundation of South Africa (grant 64787 to G. Meintjes), and the South African Medical Research Council through its TB and HIV Collaborating Centres Programme (grant RFA# SAMRC-RFA-CC TB/HIV/AIDS-01-2014, funded by the National Department of Health). This research was funded in whole, or in part, by the Wellcome Trust [098316, 214321/Z/18/Z, and 203135/Z/16/Z]. For the purpose of open access, the author has applied a CC BY public copyright license to any Author Accepted Manuscript version arising from this submission.

***Potential conflicts of interest.*** All authors: No reported conflicts. All authors have submitted the ICMJE Form for Disclosure of Potential Conflicts of Interest. Conflicts that the editors consider relevant to the content of the manuscript have been disclosed.
