# Supplementary material for: Effectiveness and Cardiac Safety of Bedaquiline-Based Therapy for Drug-Resistant Tuberculosis: A Prospective Cohort Study
Source: Clin Infect Dis. 2021 Apr 21;73(11):2083–92. doi: 10.1093/cid/ciab335 (PMC8664482; doi:10.1093/cid/ciab335)
Supplement: ciab335_suppl_Supplementary_Materials [file ciab335_suppl_supplementary_materials.docx]

**APPENDIX**

**Supplementary Methods**

Standard lead II or V5 were preferentially used for the QT measurements. However, when the end of the T wave could not accurately be determined, QTc measurement in other leads was permitted. Full blood count, chemistries, and liver function tests were performed monthly for the first six months of therapy, and then at least every six months until treatment completion. All baseline chest radiographs were assessed for the presence of cavitary lesions by a single radiologist (B.Z.).

**Supplementary Results**

Among the triplicate ECGs performed at each study visit, the mean difference between the minimum and maximum QTcF values for QTcF was 15.8 milliseconds (SD 23.4).

**Table S1: Final tuberculosis treatment outcome, overall, and stratified by HIV.**

| **TB Treatment Outcome** | **All participants**  **(n=195) (%)** | **HIV-positive**  **(n=123) (%)** | **HIV-negative**  **(n=72) (%)** |
| --- | --- | --- | --- |
| Cure | 129 (66.2) | 80 (65.0) | 49 (68.1) |
| Treatment Completion | 16 (8.2) | 11 (8.9) | 5 (6.9) |
| Treatment Failure | 8 (4.1) | 6 (4.9) | 2 (2.8) |
| Treatment interruption | 18 (9.2) | 9 (7.3) | 9 (12.5) |
| Died | 25 (12.8) | 17 (13.8) | 7 (9.7) |

P=0.61 for omnibus chi-square comparison between HIV-infected and HIV-uninfected.

**Table S2: Age strata of participants meeting primary QT-prolongation endpoints.**

| **ECG criteria** | **Age 21-30**  **(n=45, %)** | **Age 31-40**  **(n=72) (%)** | **Age 41-50**  **(n=43) (%)** | **Age >50**  **(n=23)** |
| --- | --- | --- | --- | --- |
| Any QTcF >500 ms | 0 (0) | 3 (4) | 0 (0) | 1 (4) |
| ΔQTcF >60 ms from baseline | 5 (11) | 7 (10) | 4 (9) | 3 (13) |
| BDQ temporarily stopped for QT prolongation^a^ | 2 (4) | 2 (3) | 0 (0) | 1 (4) |

^a^Four of the five participants who temporarily stopped BDQ did not have a QTcF>500 or ΔQTcF >60 ms by study ECGs. ms=milliseconds; BDQ=bedaquiline

**Figure S1: Kaplan-Meier survival curves. A, Comparing by tuberculosis drug-resistance category. B, Comparing by HIV status.**
